# GBParsy: A GenBank flatfile parser library with high speed

**DOI:** 10.1186/1471-2105-9-321

**Published:** 2008-07-25

**Authors:** Tae-Ho Lee, Yeon-Ki Kim, Baek Hie Nahm

**Affiliations:** 1 Division of Bioscience and Bioinformatics, MyongJi University, Yongin, Kyonggido, Republic of Korea; 2 Genomics Genetics Institute, GreenGene BioTech Inc., Yongin, Kyonggido, Republic of Korea

## Abstract

**Background:**

GenBank flatfile (GBF) format is one of the most popular sequence file formats because of its detailed sequence features and ease of readability. To use the data in the file by a computer, a parsing process is required and is performed according to a given grammar for the sequence and the description in a GBF. Currently, several parser libraries for the GBF have been developed. However, with the accumulation of DNA sequence information from eukaryotic chromosomes, parsing a eukaryotic genome sequence with these libraries inevitably takes a long time, due to the large GBF file and its correspondingly large genomic nucleotide sequence and related feature information. Thus, there is significant need to develop a parsing program with high speed and efficient use of system memory.

**Results:**

We developed a library, GBParsy, which was C language-based and parses GBF files. The parsing speed was maximized by using content-specified functions in place of regular expressions that are flexible but slow. In addition, we optimized an algorithm related to memory usage so that it also increased parsing performance and efficiency of memory usage. GBParsy is at least 5 - 100× faster than current parsers in benchmark tests.

**Conclusion:**

GBParsy is estimated to extract annotated information from almost 100 Mb of a GenBank flatfile for chromosomal sequence information within a second. Thus, it should be used for a variety of applications such as on-time visualization of a genome at a web site.

## Background

Large volumes of information have been rapidly accumulating since the shotgun DNA sequencing technology was introduced [1,2]. Currently, GenBank volume size is rapidly increasing, with more than 370 complete microbial genomes and over 104 assemblies of eukaryote genomes deposited over the year 2006 alone [3]. This has led to the question of how this invaluable information can be dealt with by computer programs. One of the primary concerns is how to efficiently convey huge volumes of sequence data and their annotated information to researchers. For these reasons, many sequence formats such as Abstract Syntax Notation One (ASN.1), Extensible Markup Language (XML) and GenBank flatfile (GBF) format have been used to manage sequences for distinct purposes and usages. Among these, the ASN.1 and XML format files are generally known to be difficult for a user to directly get information since the formats are designed to specify complex data structures in a machine and programming language. Thus, they are used for storing and exchanging data between computer systems. In contrast, GBF format has become one of the most popular because of its detailed sequence features and ease of readability and accessibility unlike machine-friendly ASN.1 or XML format. The format has been widely adopted to describe not only a relatively short individual gene sequence, but also long sequences, such as eukaryotic genome sequences in animals and plants [4,5]. To use the data in the file by a computer, a parsing process is required and is performed according to a given grammar for the sequence and the description in a GBF. Therefore, the GBF parser has become a routine program in bioinformatics. NCBI C/C++ toolkit [6] provides a lot of functions to deal with a sequence including parsers for ASN.1 format since NCBI employed the format as a central data format, but the toolkit does not provide a parser for the GBF. Thus, currently, several parser libraries for the GBF have been developed by other groups, such as BioPython [7], BioPerl [8] and the AJAX library in the EMBOSS package [9]. However, parsing a large GBF file (such as a eukaryotic genome sequence) with these libraries inevitably takes a long time since the parser libraries were not designed just for parsing speed. For example, parsing time for the GBF of a chromosomal sequence of *Arabidopsis thaliana *by the GBF parser of BioPython is estimated to be 109.2 seconds (Table 1). Thus, the biological community needs a faster parser that can parse a large GBF file, such as a eukaryotic chromosome, in a single-digit number of seconds at the personal computer level. We developed the GBParsy library [see Additional file 1], a C language-based parser with improved speed and efficient use of memory. In addition, we developed GBParsyPy, a GBParsy wrapper for the Python programming language.

**Table 1:** Comparative performance of GBParsy and other publicly available GBF parsers

Source	*A. thaliana*	*M. musculus*
Accession	NC_003070	NT_039207
Sequence Length (Mbp)	30.4	116.4
File Size (MB)	59.0	144.0

GBParsy	0.9 ± 0.1 (1.0)	2.4 ± 0.3 (1.0)
GBParsyPy	1.7 ± 0.1 (1.8)	3.0 ± 0.5 (1.2)
EMBOSS	9.7 ± 0.2 (10.7)	12.7 ± 0.3 (5.3)
BioPerl	45.7 ± 0.4 (50.3)	38.7 ± 0.3 (16.0)
BioPython	109.2 ± 0.4 (120.3)	39.7 ± 0.2 (16.4)

Elapsed time (sec) taken by each program was measured in 50 separate runs; mean ± standard deviation (SD) for these runs are shown. Average fold slowness in parenthesis is the ratio of each observed mean time to the one taken by the GBParsy test program.

## Implementation

In the design of the GBParsy library, we focused on the speed and the efficient use of system memory. At first, we improved parsing speed by using customized parsing functions in place of the regular expression functions. Although regular expressions are frequently used to parse strings because of their flexibility, they lower the speed of a program as a trade-off for their flexibility. Further enhancement was achieved by optimizing an algorithm related to memory usage. We tried to reduce the number of functions in the GBParsy that were for allocating new memory space and for moving data between the memory spaces, which are both time-consuming processes; thus, we were able to save system memory as well as increase performance.

The functions in GBParsy are categorized into three groups. The functions in the common group perform general operations, such as handling white spaces or lines. Each field, such as features or sequences in GBF, is handled by distinct functions included in the parsing group. The functions included in the common and parsing groups are only used internally. The functions in a user group are directly used by the user, and are implemented to handle a GBF file or parsed data. For example, the user can get a parsing result of a GBF file by calling the 'parseGBFF' function with the file name.

**Figure 1:**
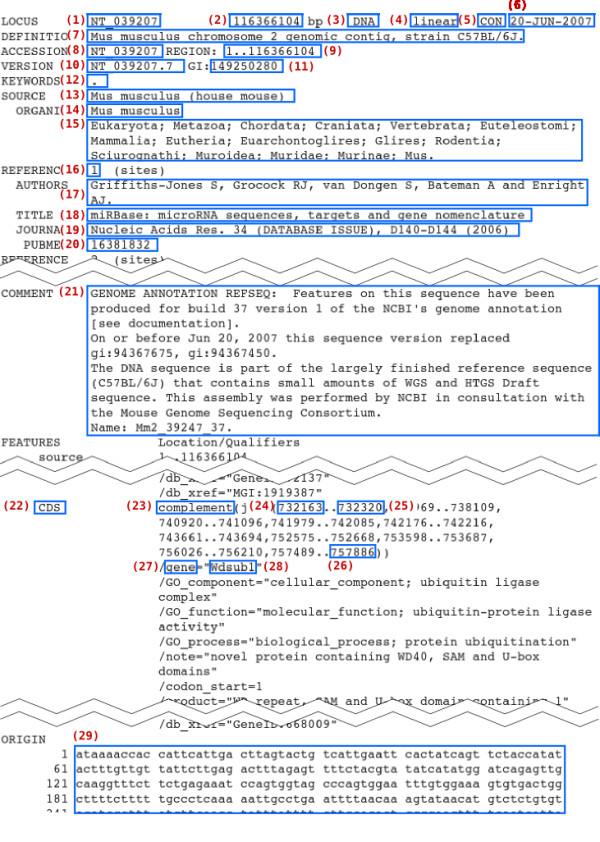
**Example of a parsing result**. Each box represents a datum as a result of parsing the GBF file, NT_039207. A number in parenthesis is denoted as such in Figure 2.

**Figure 2:**
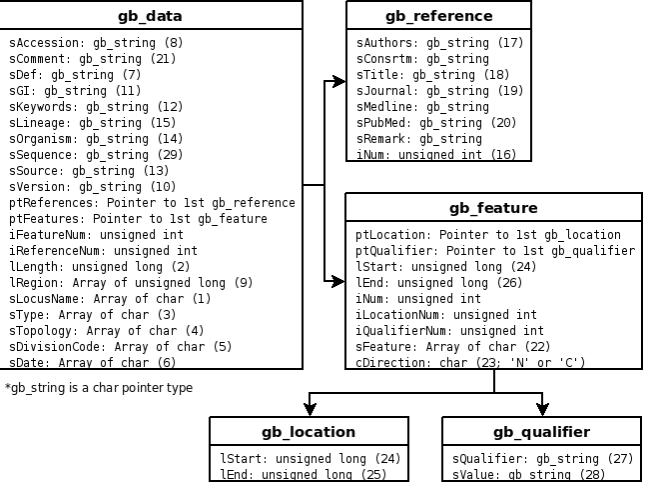
**Structures of a parsing result of GBParsy**. The parsing result of GBParsy is organized with five structures that are represented by a box. A large bold title such as gb_data represents the name of the structure and items in a box represent elements of the structure. A variable name and its data type in C language are denoted on the left- and the right-side of the colon, respectively. A number in parenthesis links with the number in Figure 1 and it represents a datum block stored in the variable.

Whole entry fields of a GBF file are parsed by calling the 'parseGBFF' function with the file name (Figure 1), and the parsing results are stored in 5 types of structures (Figure 2), which is a collection of variables and a kind of data type in C language. Gb_data structure is a main structure and a gb_data structure contains one GBF data. Each record in the reference entry and in the feature table of the GBF data is stored gb_reference and gb_feature structure, respectively. Gb_location and gb_qualifier, which is a substructure of gb_feature, contain information of a location and qualifiers, respectively. In the case of GBParsyPy, while the parser uses proper data types of the Python programming language (such as dictionary and tuple type in place of structure type of the C language), the formation of a parsing result by GBParsyPy is generally similar to that by GBParsy. For example, the main structure of GBParsy, gb_data, is implemented as a dictionary where each element in the dictionary is matched to each variable in the main structure.

## Results & Discussion

### Benchmarking test

The two largest GBF files of *A. thaliana *chromosome 1 (NC_003070) and *Mus musculus *chromosome 2 (NT_039207) were downloaded from GenBank  for the performance test. The performance of GBParsy was ascertained by benchmark testing with an Athlon 64 Processor 3200+ system with 2 GB main memory in Linux for the two publicly available chromosome sequences. To test the performance, we used an incorporated program in the EMBOSS (ver. 5.0.0) package, 'extractfeat', which parses GBF with the AJAX library and extracts specific sequences from the file. Also, we developed testing programs generating similar output with the 'extractfeat', for GBParsy, GBParsyPy, BioPerl (ver. 1.4) and BioPython (ver. 1.43), respectively. To compare the performance of each library, we extracted whole tRNA sequences from a GBF file with each testing program and determined the elapsed time. The average and standard deviation of the elapsed times (sec) for each program were measured in 50 separate runs. Slower folds for each parser were calculated against that of GBParsy to represent the performances.

### GenBank flatfile parser

Elapsed times were measured for each program in parsing two GBF files containing large chromosomal sequences and annotation of the *A. thaliana *chromosome (NC_003070) and *M. musculus *chromosome (NT_039207), (Table 1), which are known to be the largest files with proper annotation in plants and animals, respectively. When GBParsy was used, it took 0.9 sec and 2.4 sec to parse the chromosomal sequences from *Arabidopsis *and mouse, respectively. For the chromosomal sequences of *Arabidopsis*, the average fold slowness indicates that it was two orders faster than the parser of BioPython and more than 10 times faster than the AJAX parser of the EMBOSS package. In the case of the mouse chromosome sequence information, GBParsy was 16 times faster than the parser of BioPython and was five times faster than the AJAX library of the EMBOSS package. The difference in parsing performance, between the two GBF files, by GBParsy and the others resulted from percent sequence portion and/or the likewise annotation portion in the files. Since the structure of the sequence region is simpler than the annotation region of the feature fields, the difference in parsing efficiency between parsers while parsing a sequence region is small. The sequence portion of chromosomal information in *Arabidopsis *is about half of the file, while that in the mouse is over 80%. Therefore, GBParsy is more effective for a GBF file containing a large volume of annotated information than a file with poor annotation.

When the main memory is not sufficient, a computer system generally uses virtual memory, which is substantially slower than main memory. Thus, a program requiring a large volume of memory needs a long time for its execution on a system such as a personal computer. Accordingly, we tested whether the more limited use of memory by GBParsy influenced the speed of the program. This can also be estimated by measuring elapsed times. For example, when we parsed the chromosomal sequence file from mouse using a system with 512 MB main memory, the AJAX library consumed 150 seconds while GBParsy processed in eight seconds. The result reflects that GBParsy efficiently uses system memory.

GBParsyPy is a GBParsy for Python, which is a representative scripting language. Because GBParsyPy adopted GBParsy as a core parser, it inherited all of its features from GBParsy, performing at almost paralleled speed and with efficient use of memory. Thus, in the case of the *M. musculus *chromosome, GBParsyPy was over 10 times faster than the parser of BioPython or that of BioPerl. Although we did not develop a Perl version, GBParsy could be easily adopted by the language. To test usability of GBParsyPy in an application program, we developed a new program, ChrDiagram  which parses a GBF file with a parser either GBParsyPy or BioPython and draws a sequence diagram from the parsed result with GenomeDiagram [10]. GBParsyPy just took about 7 seconds in parsing the five *Arabidopsis *chromosome sequences whereas BioPython took over 5 minutes, when we drew genome diagrams of the chromosomes [see Additional file 2] with ChrDiagram at the benchmarking test system. Consequently, to draw the chromosome diagrams with full-features it took about 1 minute with GBParsyPy whereas it took over 6 minutes with BioPython. These results show that usability of application programs can be enhanced with a high speed parser.

## Conclusion

As more rapid genome sequencing technologies emerge, and more information is accumulated, the size of the GBF flatfile will be increased. As exemplified for parsing GBF files of eukaryotic chromosomes, GBParsy and GBParsyPy would be expected to significantly improve the parsing speed and the efficient use of system memory. Thus, our program would be useful for various applications that are difficult or impossible with a slow parser, such as on-time visualization of a chromosome sequence in an annotation program or web-based server.

## Availability and requirements

Project name: GBParsy (A GenBank flatfile parser library with high speed)

Project home page: 

Operating system(s): any OS that supports GCC environment

Programming language: GCC

Other requirements: Python

License: GNU GPL

Any restrictions to use by non-academics: None

## Authors' contributions

T–HL developed the parser and drafted the manuscript. Y–KK provided substantial advice and revised the manuscript. BHN provided substantial advice and guidance during all phases of the project and assisted in the drafting of the manuscript. All authors read and approved the final manuscript.

## Supplementary Material

Additional file 1 GBParsy (and GBParsyPy) version 0.60. This compressed file contains GBParsy and GBParsyPy source codes and additional files such as example programs and instruction for installation. The latest version is available at GBParsy homepage . Click here for file

Additional file 2 Diagram of *Arabidopsis *chromosome 1–5. The diagrams were drawn by ChrDiagram, in which sequence data were parsed by GBParsyPy and handled with GenomeDiagram. An orchid bar, orange red bar and olive bar on the 1st, 2nd and 3rd track denote a gene, a tRNA and a miscellaneous feature such as transposable element, respectively. Click here for file
