# Supplementary figures and images for: GBParsy: A GenBank flatfile parser library with high speed
Source: BMC Bioinformatics. 2008 Jul 25;9:321. doi: 10.1186/1471-2105-9-321 (PMC2516526; doi:10.1186/1471-2105-9-321)

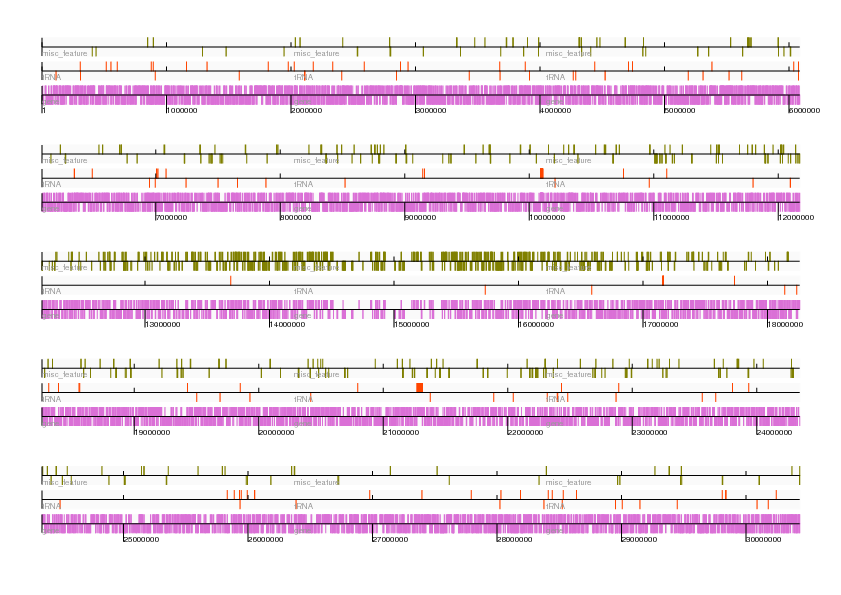

Supplement: Additional file 2 — Diagram of Arabidopsis chromosome 1–5. The diagrams were drawn by ChrDiagram, in which sequence data were parsed by GBParsyPy and handled with GenomeDiagram. An orchid bar, orange red bar and olive bar on the 1st, 2nd and 3rd track denote a gene, a tRNA and a miscellaneous feature such as transposable element, respectively. [file 1471-2105-9-321-S2.zip › Arabidopsis_Diagrams/Arabidopsis_Chr1.png]

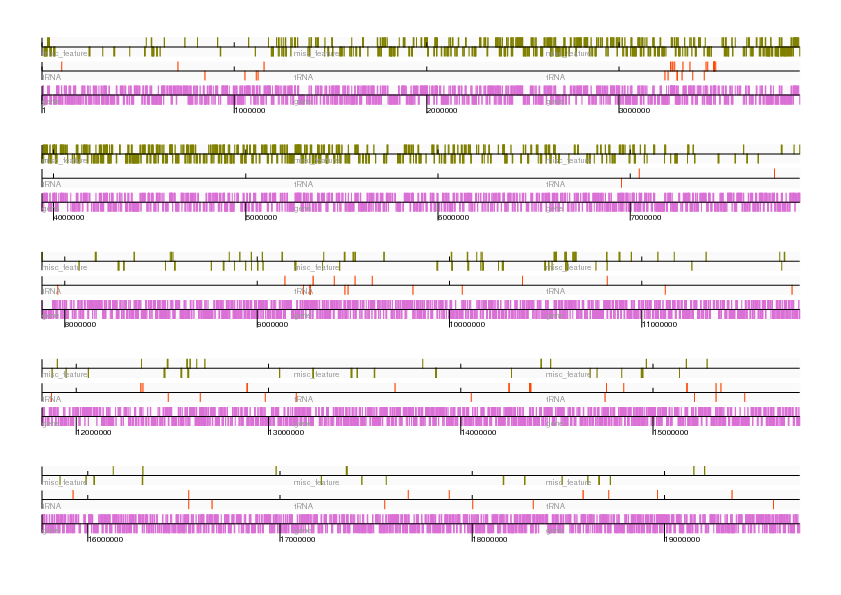

Supplement: Additional file 2 — Diagram of Arabidopsis chromosome 1–5. The diagrams were drawn by ChrDiagram, in which sequence data were parsed by GBParsyPy and handled with GenomeDiagram. An orchid bar, orange red bar and olive bar on the 1st, 2nd and 3rd track denote a gene, a tRNA and a miscellaneous feature such as transposable element, respectively. [file 1471-2105-9-321-S2.zip › Arabidopsis_Diagrams/Arabidopsis_Chr2.png]

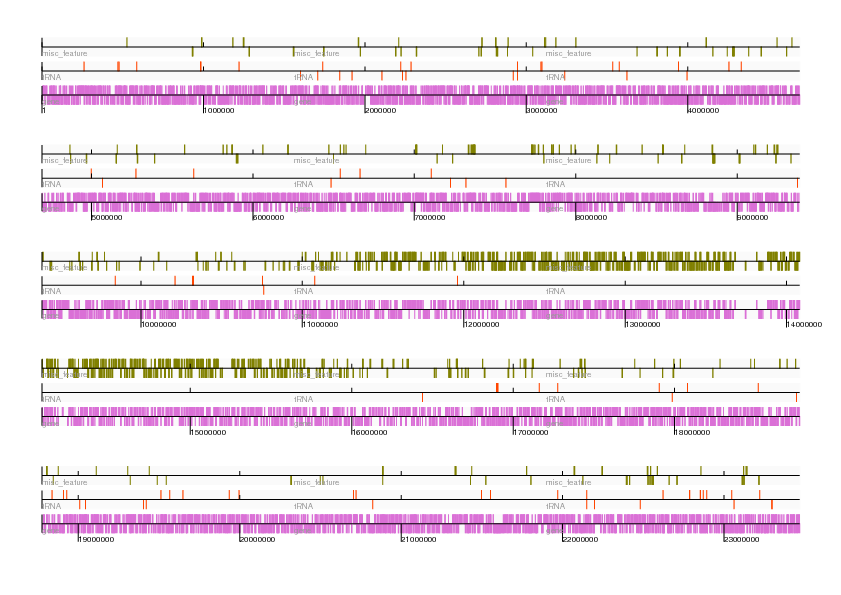

Supplement: Additional file 2 — Diagram of Arabidopsis chromosome 1–5. The diagrams were drawn by ChrDiagram, in which sequence data were parsed by GBParsyPy and handled with GenomeDiagram. An orchid bar, orange red bar and olive bar on the 1st, 2nd and 3rd track denote a gene, a tRNA and a miscellaneous feature such as transposable element, respectively. [file 1471-2105-9-321-S2.zip › Arabidopsis_Diagrams/Arabidopsis_Chr3.png]

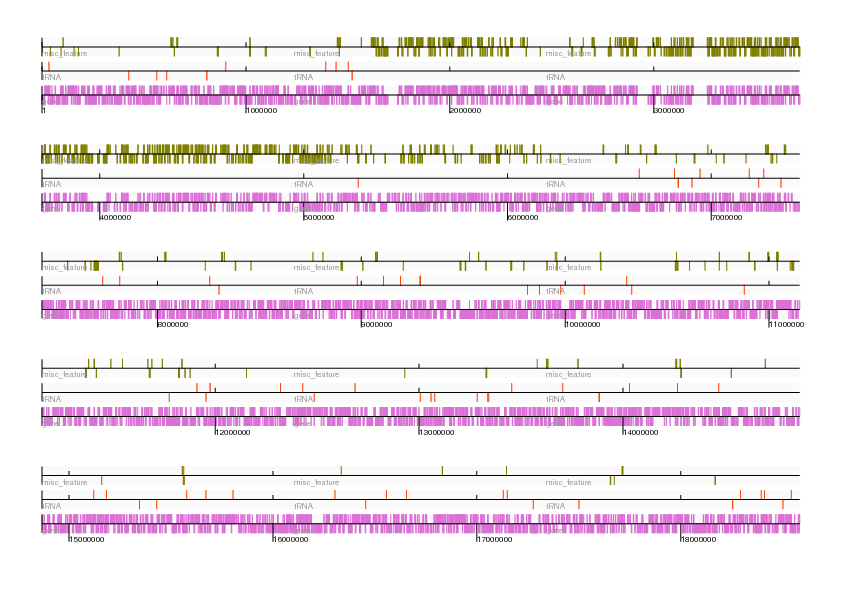

Supplement: Additional file 2 — Diagram of Arabidopsis chromosome 1–5. The diagrams were drawn by ChrDiagram, in which sequence data were parsed by GBParsyPy and handled with GenomeDiagram. An orchid bar, orange red bar and olive bar on the 1st, 2nd and 3rd track denote a gene, a tRNA and a miscellaneous feature such as transposable element, respectively. [file 1471-2105-9-321-S2.zip › Arabidopsis_Diagrams/Arabidopsis_Chr4.png]

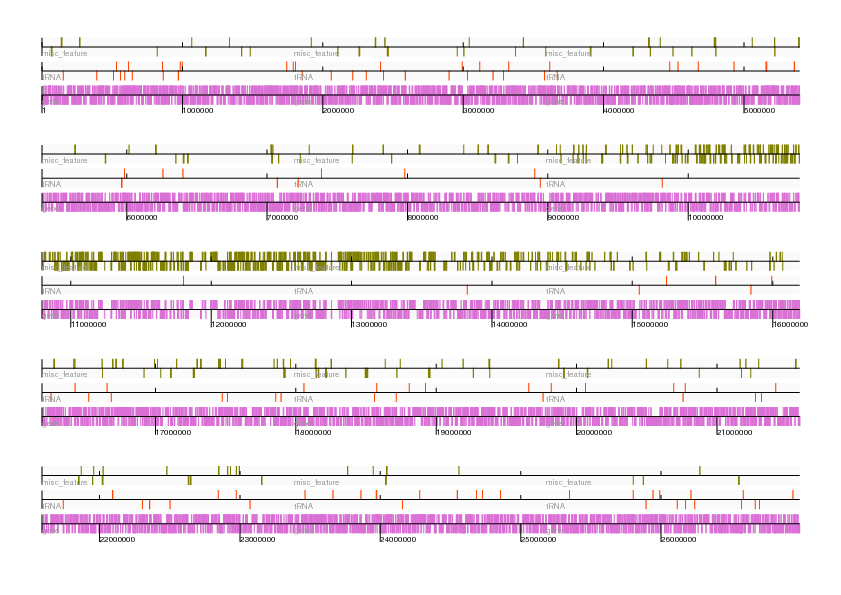

Supplement: Additional file 2 — Diagram of Arabidopsis chromosome 1–5. The diagrams were drawn by ChrDiagram, in which sequence data were parsed by GBParsyPy and handled with GenomeDiagram. An orchid bar, orange red bar and olive bar on the 1st, 2nd and 3rd track denote a gene, a tRNA and a miscellaneous feature such as transposable element, respectively. [file 1471-2105-9-321-S2.zip › Arabidopsis_Diagrams/Arabidopsis_Chr5.png]
